# Antibodies for HIV prevention: the path forward

**DOI:** 10.1002/jia2.26097

**Published:** 2023-05-17

**Authors:** Shelly Malhotra, Rachel Baggaley, Sharonann Lynch, Carmen Pérez‐Casas, Yvette Raphael, Lynda Stranix‐Chibanda

**Affiliations:** ^1^ IAVI New York USA; ^2^ World Health Organization Geneva Switzerland; ^3^ O'Neill Institute Georgetown University Washington DC USA; ^4^ Unitaid Geneva Switzerland; ^5^ Advocacy for the Prevention of HIV in Africa Johannesburg South Africa; ^6^ Harare Health and Research Consortium Harare Zimbabwe

1

The lack of accountability in defining a timely pathway for equitable access to and scale‐up of medical innovations has led to scepticism among key constituencies about the value of investing and participating in HIV prevention research. Communities are tired of promises of improved prevention options that do not materialize for those most in need.

It takes 8–10 years on average for US FDA‐approved HIV medicines to become accessible in low‐ and middle‐income countries (LMICs) [1]. The disconnect between scientific innovation and equitable impact was highlighted recently with promising data from HIV prevention trials juxtaposed against familiar delays in access in highly affected regions [2].

The topic of equitable access to innovations is often raised by those developing, financing and advocating for global health innovation when products are on the market or near licensure. At that time, high launch prices, slow plans for commercialization outside of high‐income settings and implementation challenges become apparent. But it is often too late at this stage to address the root causes of uptake barriers, and instead, public and philanthropic dollars are channelled to incrementally bridge gaps in access to products.

Potential challenges related to affordability and accessibility are compounded in the biologics space for products such as monoclonal antibodies (mAbs) that are inherently more difficult and costly to develop and produce than standard antiretrovirals (ARVs) [3]. Rather than shirk from this challenge, we must leapfrog to a new paradigm for how mAbs are conceived, developed and delivered, guided by the following four principles (Table 1):

**Table 1:** Key stakeholders and core principles for advancing mAbs access

	Discovery	Clinical development	Manufacturing and supply	Registration/policy guidance	Introduction	Scale up
Key stakeholders						
Communities and civil society	√	√	√	√	√	√
Funders	√	√	√	√	√	√
Academia	√	√				
Innovator (bio)industry	√	√	√	√		
Biosimilar industry			√	√		
Product development partnerships	√	√				
Social and implementation scientists	√	√			√	√
Procurers			√			
WHO				√		
National authorities and programmes				√	√	√
End users	√	√			√	√
Implementers						√
Recommendations						
Communities at the core	√	√	√	√	√	√
Access‐oriented research agenda—with conditions	√	√	√			
Business model innovation	√	√	√			
Embrace current tools while expanding options	√	√	√	√	√	

**Place communities at the core**: A truly person‐centred approach, with a justice and equity lens, is needed to remove systemic barriers and address asymmetries in access to mAbs [4]. Convening a civil society‐led mAbs Access Consortium might be one way to ensure accountability and foster dialogue between community networks, governments, global health actors, researchers and industry so that beneficiaries’ needs are prioritized along the development‐to‐deployment pathway.

**Embrace current tools, while expanding options**: With 1.5 million people newly acquiring HIV in 2021, we urgently need to embrace and scale up current HIV prevention tools at our disposal in a manner that prioritizes timely availability and affordable supply in high‐prevalence settings [5]. While the HIV prevention toolbox has been boosted by the recent addition of new long‐acting choices—the dapivirine vaginal ring and long‐acting cabotegravir—these options are not yet widely available globally [6].

Alongside the broadened implementation of existing options, innovation can mitigate against future loss in the effectiveness of current tools. If a highly efficacious mAb‐based product were available, this could increase choice and overcome some drawbacks of ARV‐based prevention products since mAbs do not have the potential to induce ARV resistance, which could compromise current HIV treatment programmes. Given their long‐acting profile, antibodies could also help overcome gaps in preventing peri‐ and postnatal transmission of HIV. A single birth dose of bNAbs could potentially provide months of protection during infants' period of highest transmission risk, without the same toxicity, concern of drug resistance or adherence barriers which may come from taking daily ARVs. Novel prevention trial designs are needed to ensure feasibility and address ethical considerations for trials assessing mAbs in the context of alternatives that are efficacious when used as directed, but challenging to implement in real‐world settings [7].

**An access‐oriented research agenda—with conditions**: Rather than reacting to inequities after the fact, those investing in and advancing the development of mAbs for HIV prevention should set themselves up for success from the start. A two‐part research agenda would aim to develop safe, effective, suitable mAbs products and to simultaneously invest in manufacturing capacity and innovation to make affordable production a reality by:

- *Reducing the complexity and cost of mAbs administration and supply chain* through more potent, low dose, long‐acting formulations that are temperature stable and ideally can be administered through injection, rather than intravenous infusion.
- *Driving down the cost of goods for mAbs by improving manufacturing efficiency and yield* through optimization of Chinese Hamster Ovary‐based expression systems, validation of alternative expression systems and production platforms, downstream process improvements and exploration of novel delivery approaches. The use of modular systems and single‐use bioreactors can expand the potential for manufacturing in LMICs, where HIV is most prevalent.  Technology transfer, training and capacity strengthening will be important to support this goal.

Achieving lower cost of goods could support broader access to mAbs across diseases and pathologies. Manufacturing innovations require further proof‐of‐concept studies and considerable investment to validate and bring to scale. A funding culture that rewards upstream innovation to ensure products have a target product profile and cost suitable for LMICs is needed, rather than one that mitigates downstream access inequities for ill‐suited products. This will require that all global health investments are coupled with clear and binding conditions pertaining to affordability, equitable and timely access in high‐prevalence settings, and appropriate product profile [8].

**Business model innovation**: Access to small molecule ARVs has largely relied on a business model based on transitioning a high‐price, small‐volume market into a low‐price, large‐volume, competitive market. The same levers can help drive down costs for mAbs [9]. However, unlike generic ARVs, biosimilars are not identical copies of originator mAbs and, as a result, their development is significantly more complex. The high costs associated with setting up manufacturing facilities and demonstrating comparability for mAbs can create barriers to biosimilar entry. Given the more complicated and expensive nature of mAbs development and manufacturing, a proactive strategy will be required to support the goal of affordability.

Novel business models are needed to harness market forces towards public good, enabled by voluntary licensing strategies, technology transfer, access to low‐cost reference products and waivers of additional clinical trial requirements, where appropriate [10]. To foster manufacturing efficiencies and drive down costs, one strategy could be to leverage volumes across multiple diseases and products—developing manufacturing capacity that can be applied to, and pivot between, a portfolio of different mAb‐based product candidates for endemic and epidemic diseases of regional prioritization. Building manufacturing platforms for the broader mAbs class could serve multiple diseases of LMIC or dual market relevance, subsidizing setup and production costs across a diverse disease portfolio to support manufacturing sustainability. This portfolio‐based approach could incentivize private industry to take on the ambitious task of making biologics available and accessible for a global market, instead of focusing only on a subset of the population in high‐income countries [9].

mAbs have radically improved health outcomes across diseases and show significant promise as a tool for HIV prevention. Unless the global health community acts proactively, creatively and urgently now, we will continue the status quo and lose the chance to democratize one of the best next‐generation technologies for HIV and beyond.

## 2 COMPETING INTERESTS

SM is employed by IAVI, a nonprofit organization involved in the discovery and development of mAbs.

## 3 AUTHORS’ CONTRIBUTIONS

SM wrote the Viewpoint. SM, RB, SL, CP‐C, YR and LS‐C contributed to the content, review and editing of the Viewpoint.
